# To *remove* and *replace*—a role for plasma *exchange* in counterbalancing the host response in sepsis

**DOI:** 10.1186/s13054-018-2289-1

**Published:** 2019-01-17

**Authors:** S. David, K. Stahl

**Affiliations:** 1 Division of Nephrology and Hypertension, Hannover Medical School, Carl-Neuberg-Str.1, 30625 Hannover, Germany; 2 Department of Gastroenterology, Hepatology and Endocrinology, Hannover Medical School, Hannover, Germany

Septic patients do not usually die from their infection per se but rather from an overwhelming pathological host response to it. Given that treatment is limited to resuscitation strategies, anti-infectives, and source control [1], it is of no surprise that innovative approaches that modify the overwhelming systemic reaction are highly desirable. Novel adsorption techniques have recently attracted much attention and might represent a promising avenue for further investigation [2]. In this issue of *Critical Care* Ankawi and coworkers nicely summarized the potpourri of extracorporeal techniques in septic patients ranging from (ultra) high-volume hemofiltration to modern adsorption devices and a combination of them [3]. We enjoyed reading this article and the authors are to be congratulated for their thoughtful work, in particular with regard to the balanced nature focusing not just on promises but also on pitfalls of these methods. However, given the comprehensive presentation we were surprised that a nephrologist’s old friend—which might have an underappreciated role in extracorporeal modulation of the pathological host response—seemed to be forgotten, i.e., therapeutic plasmaexchange (TPE).

The theoretical rationale for TPE goes beyond the simple (surely important) elimination of circulating injurious molecules. The exchange of septic with healthy plasma might also replace consumed protective factors that are of importance to maintain microcirculatory flow (e.g., ADAMTS13) and counterbalance vascular leak (e.g., Angiopoietin-1). About 2000 reports—mostly case reports or series—on TPE in sepsis have been published over the last 20 years**.** A recent meta-analysis identified four randomized controlled trials and found an association with reduced mortality in adults (risk ratio 0.63, 95% confidence interval 0.42 to 0.96) [4]. None of the studies were powered for survival and the cohorts were quite heterogeneous in respect to clinical severity.

Our own group has just released prospective pilot data on feasibility, safety, and secondary efficacy endpoints in early and severe septic shock patients (onset < 12 h, norepinephrine > 0.4 μg/kg/min) [5]. Based on our observation that it is feasible to recruit such severely sick patients at an early shock timepoint, an appropriately powered randomized controlled multicenter trial (NCT03065751) is currently under review for funding in Germany.

**Fig. 1:**
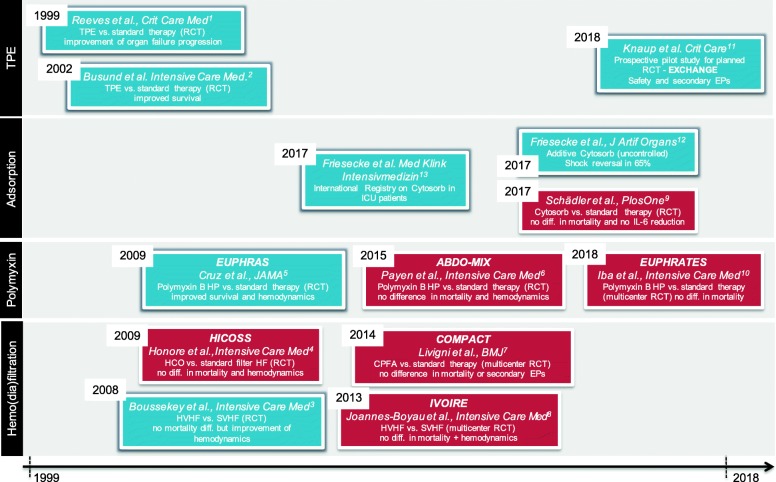
Timeline of important prospective trials of various extracorporeal therapeutic strategies in sepsis. *Abbreviations*: *TPE* therapeutic plasmaexchange, *RCT* randomized controlled clinical trial, *HP* hemoperfusion, *HVHF* high volume hemofiltration, *SMHF* small volume hemofiltration, *HCO* high cut-off, *CPFA* combined plasma filtration adsorption. References: 1. Crit Care Med. Oct;27(10):2096–104. 2. Intensive Care Med. 2002;28(10):1434–9.; 3. Intensive Care Med. 2008;34(9):1646–53. 4. Crit Care Med. 2000;28(11):3581–7. 5. JAMA. 2009;301(23):2445–52. 6. Intensive Care Med. 2015;41(6):975–84. 7. BMJ Open. 2014 8;4(1):e003536. 8. Intensive Care Med. 2013;39(9):1535–46. 9. PLoS One. 2017;12(10):e0187015. 10. JAMA 2018;320(14):1455–1463. 11. Crit Care. 2018;22(1):285. 12. J Artif Organs. 2017;20(3):252–259. 13. Med Klin Intensivmed Notfmed. 2017, doi: 10.1007/s00063-017-0342-5

Figure 1 summarizes important studies in the field of extracorporeal strategies against sepsis over the past two decades. We strongly agree with Ankawi et al. that the currently available evidence is insufficient to support the use of any extracorporeal technique in sepsis to date. Let’s change this!
